# A case report of mucocutaneous tuberculosis after orthotopic liver transplantation: a challenging diagnosis

**DOI:** 10.1186/s12879-018-3347-7

**Published:** 2018-08-29

**Authors:** Niccolò Riccardi, Giovanni Cenderello, Emanuele Borroni, Mariangela Rutigliani, Daniela Maria Cirillo

**Affiliations:** 1 Infectious Diseases and Tropical Medicine Department, Ospedale Policlinico San Martino IST, University of Genoa, Genoa, Italy; 2 Infectious Diseases Unit, Ente Ospedaliero Ospedali Galliera, Mura delle Cappuccine, 14, 16128 Genoa, Italy; 3 TB Supranational Reference Laboratory, IRCCS San Raffaele Scientific Institute, Milan, Italy; 4 Pathology Department, Ente Ospedaliero Ospedali Galliera, Genoa, Italy

**Keywords:** Cutaneous tuberculosis, OLT, HCC, LTBI

## Abstract

**Background:**

*Mycobacterium tuberculosis* is responsible for high morbidity and mortality in immune-compromised hosts.

**Case presentation:**

We present a rare case of cutaneous tuberculosis after orthotopic liver transplantation without involvement of any other organs.

**Conclusion:**

TB risk-factors assessment, careful LTBI screening and treatment according to national guidelines, as well as a reduction in missed opportunity for prevention are necessary to avoid MTB related disease in fragile patients.

## Background

Tuberculosis (TB) is principally a disease of the lungs (PTB) but, in the extra-pulmonary form (EPTB) it can affect almost any organ in the body [1].

In 2016 *Mycobacterium tuberculosis* (MTB) was responsible for 1.6 million deaths, an estimated 10.4 million people developed the disease worldwide and 15% of those had EPTB [2]. Italy is a low TB incidence country, defined by the World Health Organization (WHO) as a nation where TB frequency is lower than 10 cases per 100.000 of population [3].

EPTB is marginally responsible to the transmission of MTB but it may be difficult to diagnose, and it may remarkably contribute to TB related morbidity and lifelong disabilities [4, 5];

it is otherwise difficult to be recognised and diagnosed. In addition, cutaneous tuberculosis (CTB), due to either direct inoculation from an exogenous source, from an endogenous source or due to haematogenous dissemination of MTB, is a rare manifestation, accounting for 1 to 1.5% of all EPTB. Due to MTB heterogeneous manifestations, its diagnosis may be problematic in European countries where TB incidence is low [2–6].

For rifampicin susceptible TB, a 2 months, induction phase with oral isoniazid (H), rifampicin (R), ethambutol (E) and pyrazinamide (Z) is followed by 4 months of maintenance with oral rifampicin and isoniazid is needed to manage and eventually cure CTB; in few cases surgical removal of the lesion may be considered [6].

In this report, we are presenting a case of a patient who underwent orthotopic liver transplantation (OLT), due to hepatocellular carcinoma (HCC), 14 months before clinical presentation of CTB. The patient was under immunosuppressive treatment with both tacrolimus and everolimus when he developed CTB of the lower lip. We will also discuss optimum clinical management of CTB.

## Case report

**Fig. 1:**
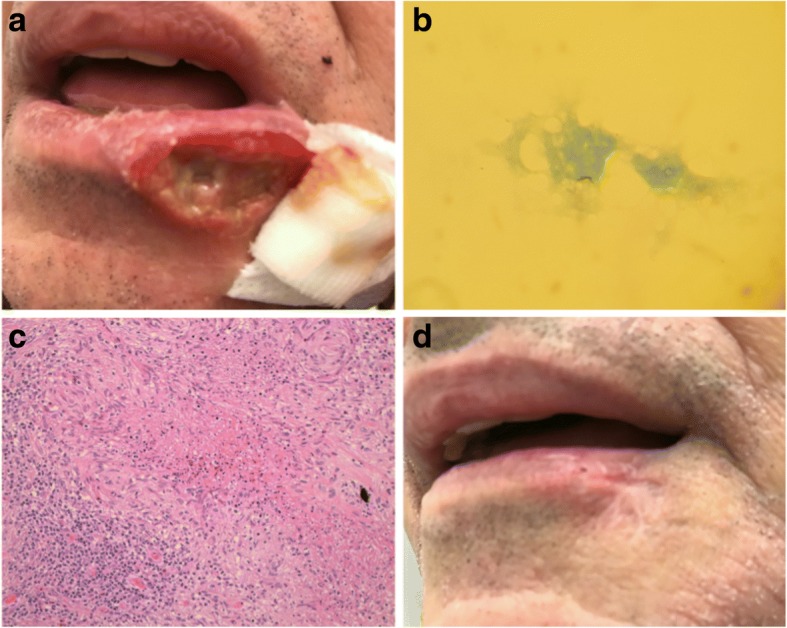
**a** Ulcerative lesion of the left lower lip as it appears at the first clinical visit at our outpatients department. **b** Ziehl-Neelsen staining showing Acid fast bacilli (AFB) in the patient’s sample. Smear scored 1+. **c** Subcutaneos granuloma: degenerate collagen is surrounded by a palisade of hystiocytes, lymphocites, fibroblasts and note of intensely eosinophilic necrobiosis (E&E × 20). **d** Complete resolution of the lesion after 6 month of treatment

A 59-year-old Italian male, weighting 69 kg and 173 cm tall, came to our attention for an ulcerative lesion of the left lower lip (Fig. 1, Panel A). He had already received antibiotic treatment with amoxicillin/clavulanate plus antiviral acyclovir for 10 days in other outpatient facilities without any clinical improvement. His clinical history was remarkable for hepatitis B (HBV) and Genotype 3 hepatitis C (HCV) co-infection, which led to OLT due to HCC, and several years spent in foreign countries. In fact, when he was in his late 40 he had spent 6 years in Nigeria and one and a half year in the South of China where he worked at sea as a kitchen supervisor. The patient was HIV negative. Six months before the OLT he had received treatment with daclatasvir (60 mg/die), sofosbuvir (400 mg/die) and ribavirin (1000 mg/die) for HCV, successfully reaching sustained virological response (SVR) 12 weeks after the end of treatment. A QuantiFERON®-TB Gold In-Tube (QFT-G) was performed among the pre-transplant screening and resulted positive.

Neither before nor after OLT, latent TB infection (LTBI) therapy was administered.

Patient received OLT and 14 months post-transplant presented with a lower lip lesion. At the time of presentation patient was on the following medications: entecavir1000 mg daily for chronic HBV with lamivudine resistance, tacrolimus 3 mg daily and everolimus 1 mg twice a day for immunosuppression.

A punch biopsy of the lower lip lesion was performed and submitted for extended microbiology and histological examination.

The histological examination suggested chronic granulomatous inflammation (Fig. 1, Panel B).

Real time PCR (Xpert MTB/Rif™^–^ Cepheid Sunnyvale, CA United States) was positive for MTB by high grading, implying a high bacterial load in the analysed specimen. No rpo-B mutation, affecting rifampicin resistance, was detected. Conventional microbiological investigations were also carried out: smear microscopy and automated liquid cultures (Bactec MGIT960™– Becton and Dickinson Franklin Lakes, NJ) were positive and the subsequent susceptibility testing showed sensitivity to all first-line drugs tested. A total body CT scan was performed to rule out presence of granuloma or signs of pulmonary or other extra-pulmonary site involvement. Moreover, Xpert MTB/Rif™^–^, Ziehl Neelsen and MTB colture on sputum resulted negative.

A treatment with rifabutin (450 mg/daily), isoniazid (300 mg/daily), ethambutol (1200 mg/daily), pyrazinamide (1500 mg/daily) and daily supplementation of B6 vitamin was started for the intensive phase of 2 months. The therapeutic regimen was then simplified to rifabutin (300 mg/daily) plus isoniazid (300 mg/daily) for the following 4 months.

Liver function and level of immune-suppressive treatment were monitored weekly. No increase in transaminases was observed and only a slight decrease in both tacrolimus (from5 μg/L to 3; normal value 5–7 μg/L) and everolimus (from 3 μg/L to 1.9 μg/L; normal value 2.5–3 μg/L) was noticed after 1 month of treatment; therefore, to achieve satisfactory blood-level concentrations, tacrolimus dosage was increased to 6 mg/ daily and everolimus was progressively titrated to 2.75 mg/ daily in two doses. After 3 weeks of therapy, a dramatic clinical improvement was observed and after 6 months of treatment the lesion was cured (Fig. 1, Panel D).

## Discussion and conclusion

This case highlights a rare localisation of TB, without pulmonary or other organs involvement, in a chronic immunosuppressed host. Prior to transplant, a detailed anamnesis is necessary to estimate the risk of LTBI and/or active TB. In fact, even if our patient was born and raised in a low TB prevalence country, he lived and worked for several years, in high TB prevalence nations [2].

Subsequently screening for latent TB in patients undergoing OLT and other solid organ-transplant (SOT) is mandatory to assess the risk of developing active-TB and planning LTBI treatment or prophylaxis [7, 8]. As suggested by Zenner et al., either TST or IGRA-test may be used to annually monitor candidates to SOT [9]. In this case, the patient had a positive IGRA-test and, although drug related toxicity may be a concern, the benefit of LTBI treatment in a host undergoing chronic-immunosuppression should drive the choice. Furthermore, a 3 months isoniazid-rifampicin dual regimen has been proven to be safe and efficient [10]. Unfortunately, due to fear of liver toxicity and possible drug-drug interactions, treatment of LTBI before and after SOT was postponed.

Although CTB diagnosis is often overlooked due to the variety of possible differential diagnosis, the molecular diagnostic tools for TB detection approved by WHO, remarkably reduce time to diagnosis and are both sensitive and specific [11, 12].

CTB often represents the haematogenous or lymphatic spread of MTB from other foci therefore, treatment of sensible CTB follows the same rules of TB of other organs with an intensive phase of 2 months with isoniazid, rifampicin, ethambutol and pyrazinamide followed by a maintenance phase of 4 months with isoniazid and rifampicin. Thus, when CTB is suspected, a diagnostic approach aimed to exclude internal organ involvement, especially PTB, is mandatory.

Rarely CTB is confined as only cutaneous, probably for direct inoculation of MTB, like in the case of tuberculosis verrucosa cutis and lupus vulgaris: these forms of CTB are not associated with internal organ involvement but, the length and the regimen of sensible MTB treatment remain the same [12].

Treatment with rifabutin was initiated to decrease the chance of drug to drug interactions due to the use of molecules which share the same cytochrome P 450 (CYP450) metabolic pathway. Rifampicin is a metabolic inducer of CYP450 and may decrease calcineurin inhibitors haematic level [13, 14]. Rifabutin, as a milder inducer of CYP450, may have a lower impact on both calcineurin inhibitors then rifampicin [14]. Therefore, according to previous studies that compared rifamycins in TB treatment, rifabutin was started along with careful monitoring of both tacrolimus and everolimus in order to decrease the likelihood of modifications in calcineurin inhibitors haematic level [15–18].

A clinical relevant reduction in tacrolimus and everolimus blood levels was then expected, but the extent was not predictable [14]. In addition, all drugs used for the CTB are known to potentially cause liver toxicity, undermining the treatment tolerability, OLT and clinical outcome. However, different reports described efficacy and safety of rifabutin in treating TB patients who presented allergy to rifampicin or potential harmful drug-drug interactions [15].

Finally, cost-effectiveness of TB screening is also an important issue. In Italy different protocols are in use to screen categories at risk. TST is often used as first screening because of the very low cost and in some referral medical centres positive cases are confirmed by IGRAs. IGRAs sensitivity has been reported higher then TST in several studies in HIV infected individuals [19]. In any case the intention to test should be linked to the intention to treat or, if treatment is not possible, to monitor the patient, particularly if the immunity is impaired.

As suggested by the TBNET consensus, treatment of LTBI in transplant candidates and after SOT should be offered according to national guidelines, thus reflecting regional drug availability and resistance patterns [20]. However, if LTBI treatment is not possible both during pre-transplant phase and in the post-transplant phase, close monitoring of signs and symptoms of active TB is recommended [20]. In patients undergoing SOT in low TB prevalence countries, careful anamnesis, TB risk factors assessment and LTBI screening are recommended. Although a rare presentation of active TB, due to the plethora of cutaneous presentation, CTB should be suspected in immune-compromised host.

Molecular tests and classical microbiological investigations on bioptic specimen are helpful tools to facilitate correct diagnosis of CTB.

## Abbreviations

CTB: Cutaneous Tuberculosis
CYP 450: Cytochromes P450
E: Ethambutol
EPTB: Extra-pulmonary Tuberculosis
H: Isoniazid
HBV: Hepatitis B virus
HCC: Hepatocellular carcinoma
HCV: Hepatitis C virus
HIV: Human Immunodeficiency Virus
IGRA: Interferon Gamma Release Assay
LTBI: Latent TB infection
MTB: Mycobacterium tuberculosis
OLT: Orthotopic liver transplantation
P: Pyrazinamide
PTB: Pulmonary Tuberculosis
QFT-G: QuantiFERON®-TB Gold In-Tube
R: Rifampicin
SOT: Solid organ-transplant
SVR: Sustained virological response
TB: Tuberculosis
TST: Tubercolin skin test
WHO: World Health Organization
